# An Emergency Department Survey on Research Participation in the Patient With Suicidal Ideation or Suicide Attempt

**DOI:** 10.1177/23743735231218866

**Published:** 2023-12-06

**Authors:** Kevin Skoblenick, Marissa Hsu, Jennifer Swainson

**Affiliations:** 1 Department of Emergency Medicine, University of Alberta, Edmonton, AB, Canada; 2 Department of Emergency Medicine, Royal Alexandra Hospital, Edmonton, AB, Canada; 3 Neuroscience and Mental Health Institute, 12357 University of Alberta, Edmonton, AB, Canada; 4 3146 Alberta Health Services, Edmonton, AB, Canada; 5 Department of Psychiatry, University of Alberta, Edmonton, AB, Canada; 6 Department of Psychiatry, Misericordia Community Hospital, Edmonton, AB, Canada

**Keywords:** Suicide, suicidality, emergency department, survey

## Abstract

The patient expressing suicidal ideation is a common mental health presentation in the emergency department (ED) but a notion exists that this psychiatric crisis precludes them from research participation. In order to better understand the potential research participation of suicidal patients, this study surveyed patients in the ED with suicide attempt or ideation. This was an anonymized survey study interviewing 50 patients in a tertiary care ED with a chief complaint of suicide attempt or suicidal ideation. A script was read by the research assistant regarding a hypothetical research study using ketamine to treat suicidality in the ED and asked to rate their interest in participation in the study as well as their interest in a 1-week follow-up call. Most patients (84%) reported that they would be interested in participating in this research project while 96% of all 50 patients would be interested in a follow-up phone call at 1 week. The data from this study should help other emergency medicine and psychiatry researchers advance projects in this underserved ED patient population.

## Key Points

- Suicidal ideation and suicide attempt is a common emergency department (ED) presentation with minimal research studies addressing it potentially due to a stigmatized view of this patient population.
- Our survey study of suicidal patients in the ED showed that 84% of patients were interested in participating in a research study while 96% were interested in a follow-up phone call 1 week after their ED visit.
- These results highlight that a patient's mental health crisis should not be a barrier to having a discussion about research participation.

## Introduction

Suicide is the second leading cause of death in Canadians aged 15–34 years old and remains one of the top five causes of death until the age of 60.^1^ Suicidal ideation (SI) often precedes death by suicide and is a common mental health presentation to the emergency department (ED). Research to support improved treatment of these patients in the ED is also limited, perhaps due to a preconceived notion that these patients will be unwilling to engage in a research study due to their mental health crisis.^2^ It has also been suggested that these patients may be too distressed and that any such research into suicidality should be restricted to the outpatient clinic setting.^3^ As a result of these presumptions, researchers may face more barriers during the institutional ethics board review of projects related to patients with SI. This unfortunate prolonged ethics board review may ultimately be a deterrent for those interested in this field and further decrease the research being performed in this area.

While there has been a paucity of treatments available specifically for the symptom of SI, a single dose of intravenous ketamine (IVK) has been shown to reduce suicidality in an outpatient setting.^4,5^ It would follow that this may be a beneficial and viable treatment option in the ED. As a precursor to further work to investigate efficacy of IVK in the ED patient with SI, this brief interview study surveyed this patient population to gauge their interest in participating in a randomized control trial (RCT) should one be available.

## Methods

This was a convenience sample interview study obtained during hours patients have been clinically observed to commonly present with SI (2000–2300 hours). Interviews occurred between February 2023 and May 2023.

The interviews were conducted in a tertiary care ED in Edmonton, Alberta with approximately 80,000 ED visits annually. The ED has a dedicated mental health area staffed by trained mental health nurses. Adult (18+) patients presenting with SI were identified by the treating mental health nurses as a patient who had presented with SI or suicide attempt after they were medically cleared by the ED physician. This was almost always within 2–6 hours of the patient arriving to the ED but rare cases may have been delayed (10–12 hours) due to a prolonged medical clearance period.

Once patients were identified a separate, research-trained ED nurse dedicated to this interview study approached the patient. After obtaining consent to speak with the patient regarding this survey and informing patients that the survey would not affect their current treatment, the ED nurse described a hypothetical research study involving a potential ED intervention for suicidality (low dose ketamine infusion). The survey script (Appendix S1**)** described that this is an intervention with some data suggesting a limited time efficacy for treating the symptom of SI and that it would not work for all patients. Additionally, the survey nurse described the structure of an RCT, including potential for enrollment in the placebo arm. The patient was then asked two questions:

- “If this study were available today, how interested would you be in participating on a scale from 1 (no interest at all) to 7 (definitely interested)?”
- “If this study were available today, how interested would you be to follow-up with a health provider at 7 days, on a scale from 1 (no interest at all) to 7 (definitely interested)?”

Answers to these questions, as well as patient age and gender (male, female, or other) were recorded in REDCap survey software (Nashville, TN). Verbal informed consent was obtained from the patients for their anonymized responses to be used for this study.

## Results

**Figure 1.**
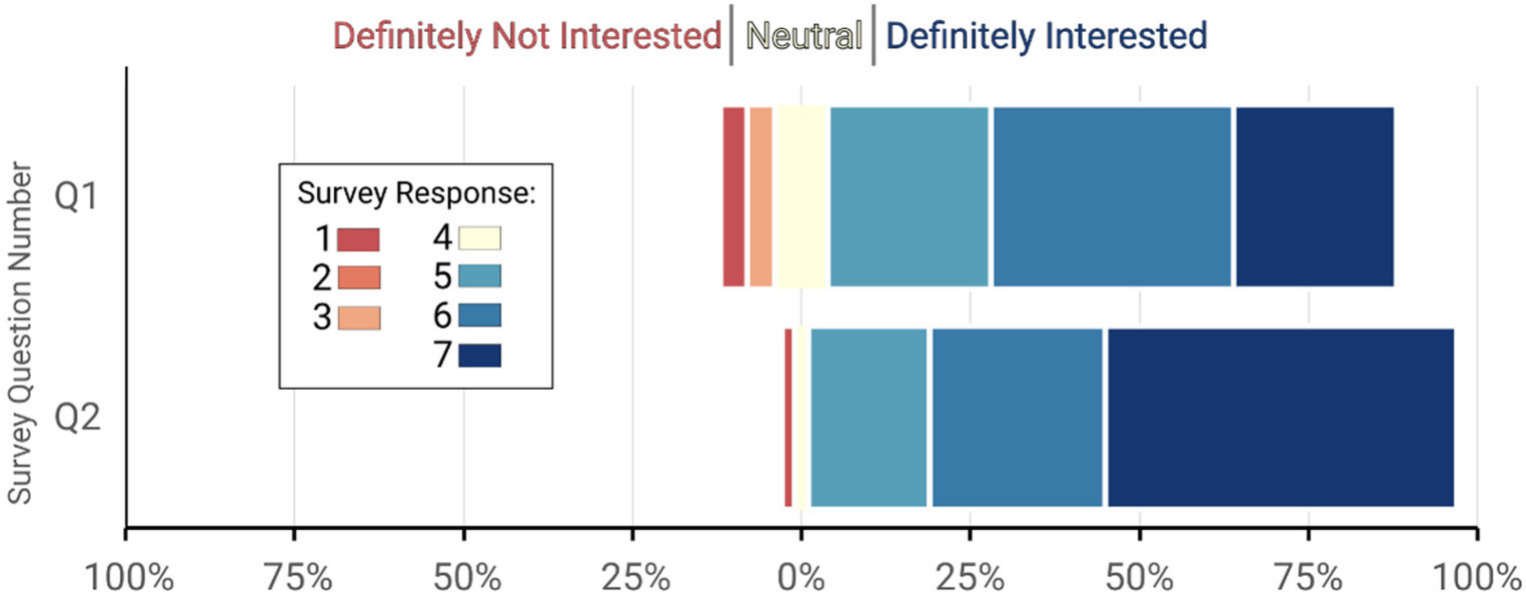
Survey responses to the questions: Q1: “If this study were available today, how interested would you be in participating on a scale from 1 (no interest at all) to 7 (definitely interested)?” Q2 – “If this study were available today, how interested would you be to follow-up with a health provider at 7 days, on a scale from 1 (no interest at all) to 7 (definitely interested)?.”

A total of 51 patients’ ages 18–79 were approached by the research nurse over 12 weeks. One patient declined to hear the survey script or participate further, and the other 50 (22 male, 28 female) completed the survey. An overwhelming majority (84%) reported that they would be interested to participate in a research study such as this (Figure 1). Further, all but 2 patients (96%) indicated interest in a follow-up phone call 1 week from their ED visit regarding the research project should they be enrolled in this hypothetical study.

## Discussion

Despite the mental health crisis they may be experiencing, ED patients with SI reported a positive interest to take part in a research study to investigate a potential treatment for SI. That 50 out of 51 patients approached were interested in hearing the survey regarding the potential study also highlights the underlying eagerness to for this patient group to be involved in research. These patients also expressed a general interest in a follow up call, as part of any potential study. This is important in assessing whether any intervention for SI in the ED, such as an IVK infusion results in both immediate and more prolonged resolution of SI. Results of this survey support feasibility of patient participation in research.

## Limitations

By using a convenience sample and only interviewing patients during their most common presentation time this study may have inadvertently isolated a subset of patients with SI or attempt. Additionally, this survey asked participants about a specific study (IVK) while interest in general research participation may be lower if there isn’t a specific treatment being investigated. The survey script explicitly highlighted that the theoretical treatment is not being offered today nor is it a guaranteed efficacy. It also reviewed the side effects and time limited effect of IVK. Despite these efforts, there is a risk that patients may have conflated their desire to participate in the study with a desire for an intervention that would alleviate their suicidality. Future studies using interventions for this population may have slight differences in the patient interest level however it would not be expected to deviate significantly.

## Conclusion

As risks exist in studying a patient population at risk of self-harm and suicide, appropriate informed consent and ethics approval for any future studies is essential. However, due to potential impact on patient care and demonstrated willingness of this patient population, studies on interventions, particularly ones with previously demonstrated efficacy in the outpatient population, should be pursued. In order to improve the care of these patients and identify potential treatment options, researchers should actively engage in the suicidal ED patient to explore interventions which may be of benefit.

## Supplemental Material

sj-pdf-1-jpx-10.1177_23743735231218866 - Supplemental material for An Emergency Department Survey on Research Participation in the Patient With Suicidal Ideation or Suicide Attempt Click here for additional data file. Supplemental material, sj-pdf-1-jpx-10.1177_23743735231218866 for An Emergency Department Survey on Research Participation in the Patient With Suicidal Ideation or Suicide Attempt by Kevin Skoblenick, Marissa Hsu and Jennifer Swainson in Journal of Patient Experience

## References

[1] Table: 13-10-0394-01 Leading causes of death, total population, by age group Table: 13-10-0394-01 (2018).

[2] Petrik ML Gutierrez PM Berlin JS Saunders SM . Barriers and facilitators of suicide risk assessment in emergency departments: a qualitative study of provider perspectives. Gen Hosp Psychiatry. 2015;37(6):581-6. doi:10.1016/j.genhosppsych.2015.06.018 26208868

[3] Oquendo MA Porras-Segovia A . Barriers for the Research, Prevention, and Treatment of Suicidal Behavior. In: Baca-Garcia E, ed. *Behavioral Neurobiology of Suicide and Self Harm*. Springer International Publishing; 2020:25-40. *Current Topics in Behavioral Neurosciences*. 10.1007/7854_2020_159 32986218

[4] Witt K Potts J Hubers A , et al. Ketamine for suicidal ideation in adults with psychiatric disorders: a systematic review and meta-analysis of treatment trials. Aust N Z J Psychiatry. 2020;54(1):29-45. doi:10.1177/0004867419883341 31729893

[5] Fu DJ Ionescu DF Li X , et al. Esketamine nasal spray for rapid reduction of major depressive disorder symptoms in patients who have active suicidal ideation with intent: double-blind, randomized study (ASPIRE I). J Clin Psychiatry. 2020;81(3). doi:10.4088/JCP.19m13191 32412700

