# Supplementary material for: An Emergency Department Survey on Research Participation in the Patient With Suicidal Ideation or Suicide Attempt
Source: J Patient Exp. 2023 Dec 6;10:23743735231218866. doi: 10.1177/23743735231218866 (PMC10704937; doi:10.1177/23743735231218866)
Supplement: sj-pdf-1-jpx-10.1177_23743735231218866 - Supplemental material for An Emergency Department Survey on Research Participation in the Patient With Suicidal Ideation or Suicide Attempt [file sj-pdf-1-jpx-10.1177_23743735231218866.pdf]

Script for ED Interview Regarding Ketamine Treatment for Suicidality
Hi <patient name>.
I am <name> and am a research assistant here in the Royal Alexandra Hospital Emergency Department. You have been identified as someone who presented to the emergency department with suicidal thoughts. Is that correct? If so, would you be willing to talk to me about a future research project we are planning for people with these thoughts?
[If no]
Thank you for your time.

[If yes]
I am going to describe a hypothetical research study for patients with thoughts of suicide/self-harm. At the end I will ask you two questions and record your age and gender. No other information regarding you will be recorded. Is this OK?

[If yes]
There is no research study happening now. (pause) Today, I would like to describe a study that we might do in the future. We are trying to find how our many patients like you would be interested to participate if it was available.

The study would involve a treatment that might temporarily help to reduce thoughts of suicide. This treatment has been used in emergency departments around the globe for caring for patients with injuries and headaches; however, there is now an interest in testing it in patients with thoughts of suicide/self-harm.

People who participate in the study would be given a medicine given through an intravenous route over a 40 minute period. This would either be the active medication called ketamine or something called a placebo, which is a nonactive treatment. This approach is used so that we can determine the true effect of the medication. Those involved in the study would need to be closely monitored during and after the IV infusion for a few hours.

The study would also require participants to complete questionnaires during their time in the emergency department that assess how they are feeling with regards to thoughts of suicide. All medication have the potential to produce side effects. The most common types would include small increases in high blood pressure, nausea, vomiting, and anxiety. These symptoms are usually brief and resolve within 2 hours. The beneficial effects of this treatment are variable in all patients and may only last for 5-7 days.

Question #1: If this study were available today, how interested would you be in participating on a scale from 1 (no interest at all) to 7 (definitely interested)?

Question #2: If this study were available today, how interested would you be to follow-up with a health provider at 7 days, on a scale from 1 (no interest at all) to 7 (definitely interested)?

[**Question #1**: **Record 1-7 response**]: __
[**Question #2**: **Record 1-7 response**]: __
[Record age: __ __]
[Record stated gender: M / F / O]
Thank you for your time.
